# Supplementary figures and images for: Structures of the Hepaci-, Pegi-, and Pestiviruses envelope proteins suggest a novel membrane fusion mechanism
Source: PLoS Biol. 2023 Jul 11;21(7):e3002174. doi: 10.1371/journal.pbio.3002174 (PMC10335668; doi:10.1371/journal.pbio.3002174)

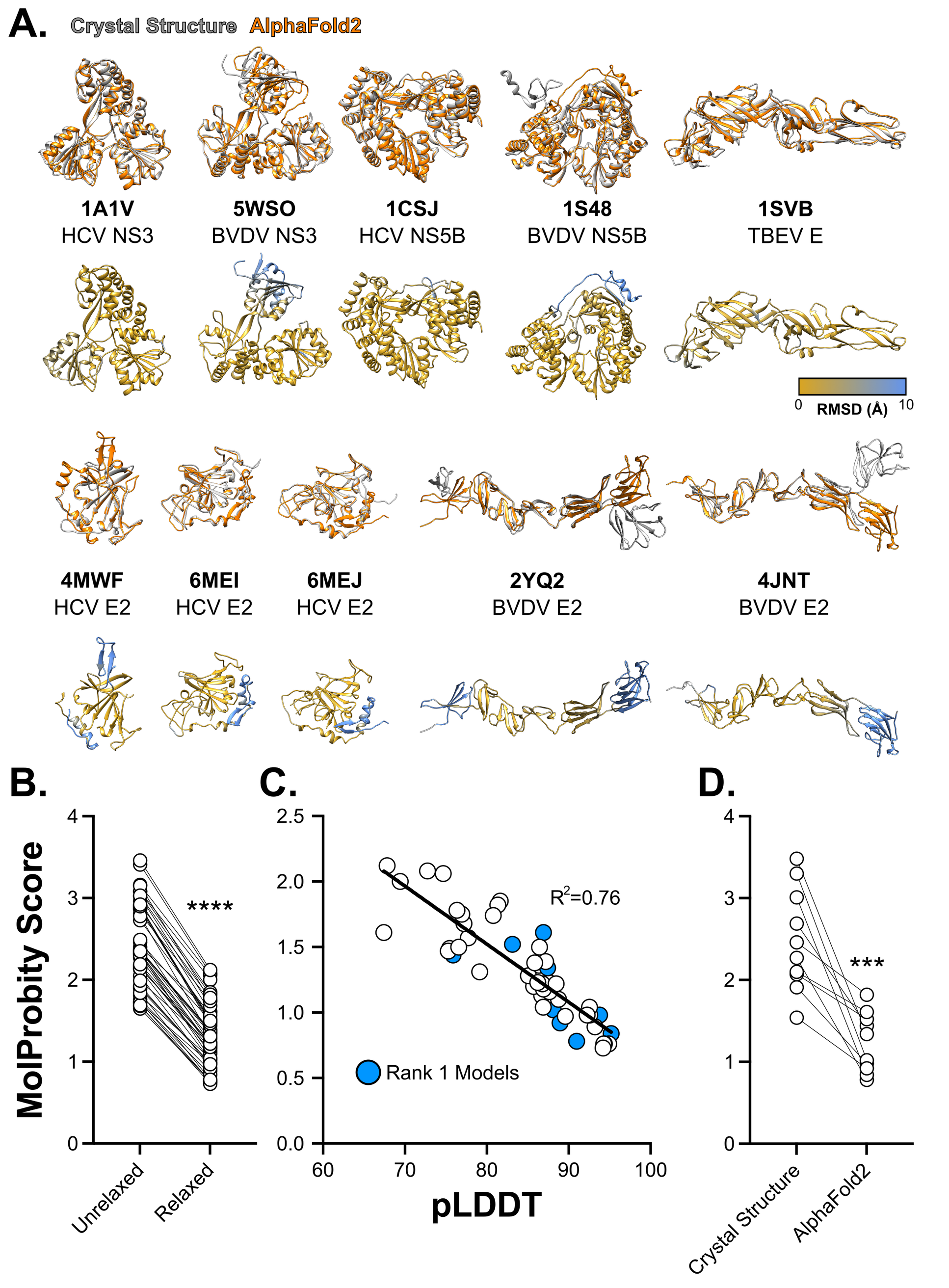

Supplement: S1 Fig — (A) AlphaFold predicted structures superposed with their cognate experimental structure from the protein database (PDB codes are provided for each structure; TBEV, tick-borne encephalitis virus). In each case, the lower structure represents the AlphaFold model colour coded by RMSD from its experimentally determined partner structure. Blue indicates disagreement, as denoted in the colour key. (B) MolProbity scores of all AlphaFold benchmarking models before and after AMBER relaxation. Each data point represents an individual model, n = 50 (5 candidate models per viral target). Lower values indicate higher model quality. (C) AlphaFold model confidence plotted against MolProbity score for all benchmarking structures (n = 50, with rank 1 models shown in blue). The negative correlation indicates higher model quality with higher confidence scores (linear correlation). (D) Comparison of MolProbity scores for AlphaFold structures and their cognate experimental structures. Asterisks indicate degree of statistical significance (t test). Underlying numerical data are available in S1 File. Further description can be found in S1 Text. (TIF) [file pbio.3002174.s004.tif]

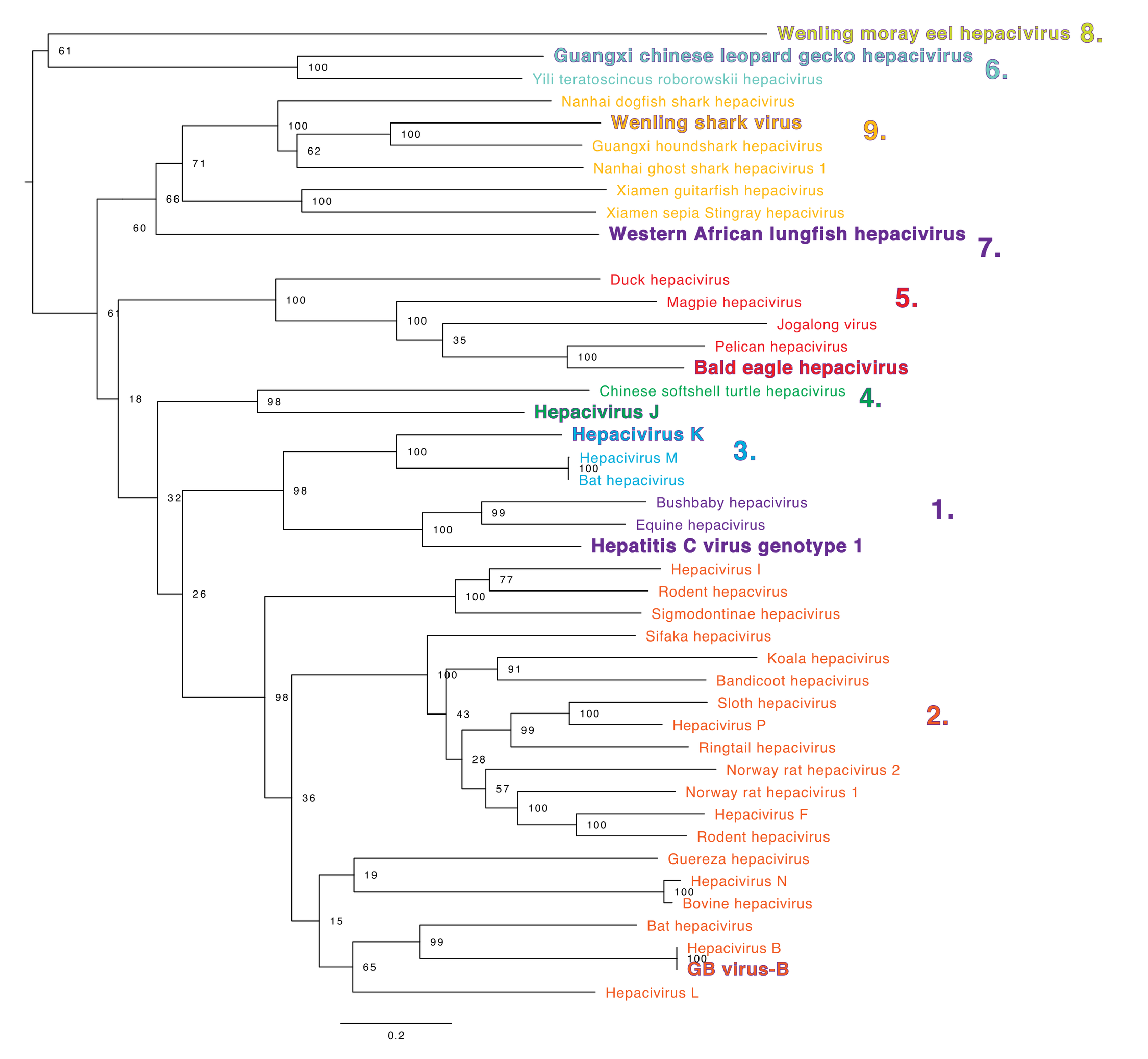

Supplement: S2 Fig — Subclades are numbered and colour coded; species in bold text represent clade-specific reference viruses. E1 and E2 annotations from these reference viruses were propagated throughout aligned whole genome sequences of each subclade. Further description can be found in S1 Text. (TIF) [file pbio.3002174.s005.tif]

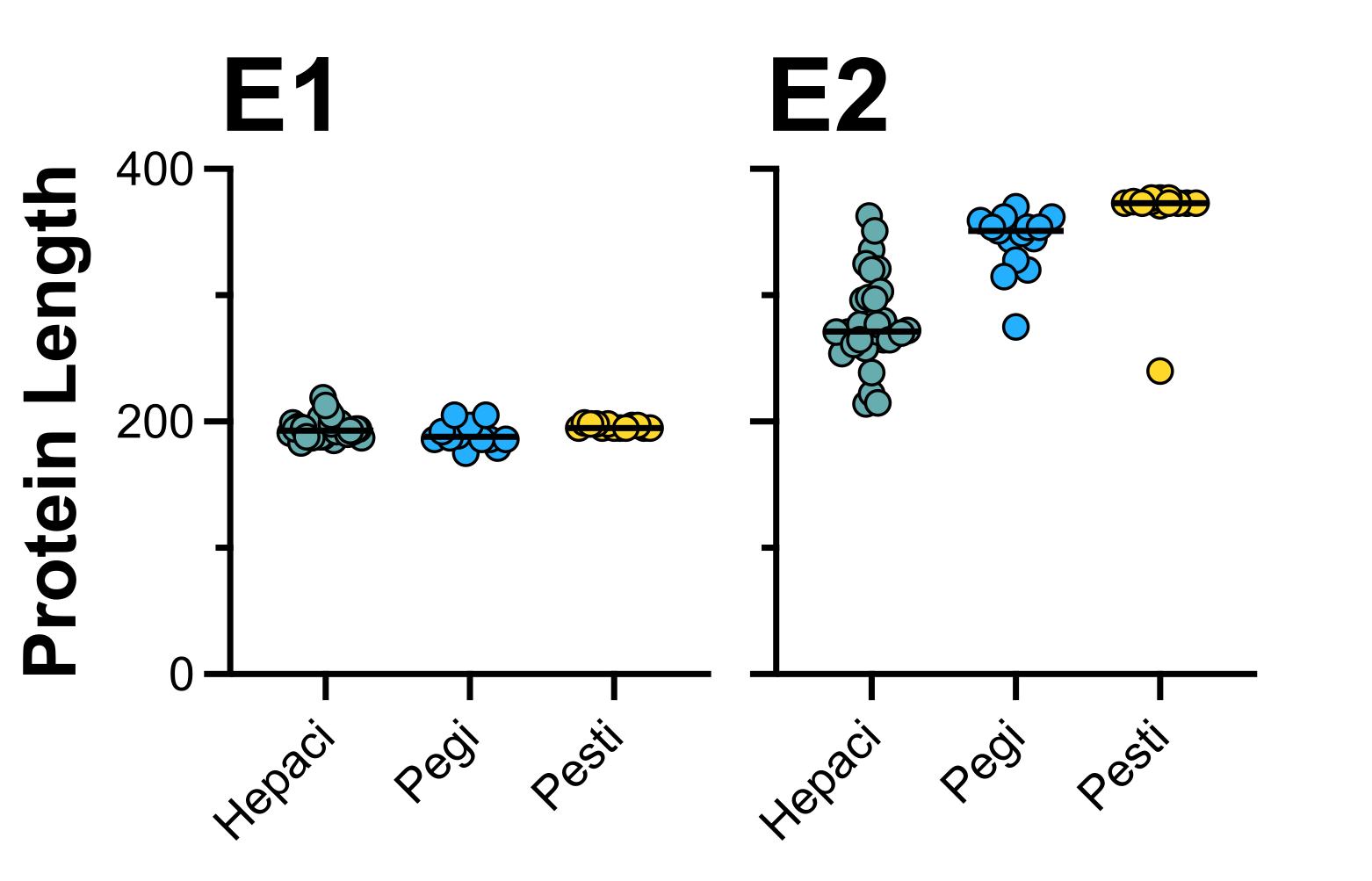

Supplement: S3 Fig — Each data point represents an individual viral species, n = 32, 15, and 13, respectively. Underlying numerical data are available in S1 File. Further description can be found in S1 Text. (TIF) [file pbio.3002174.s006.tif]

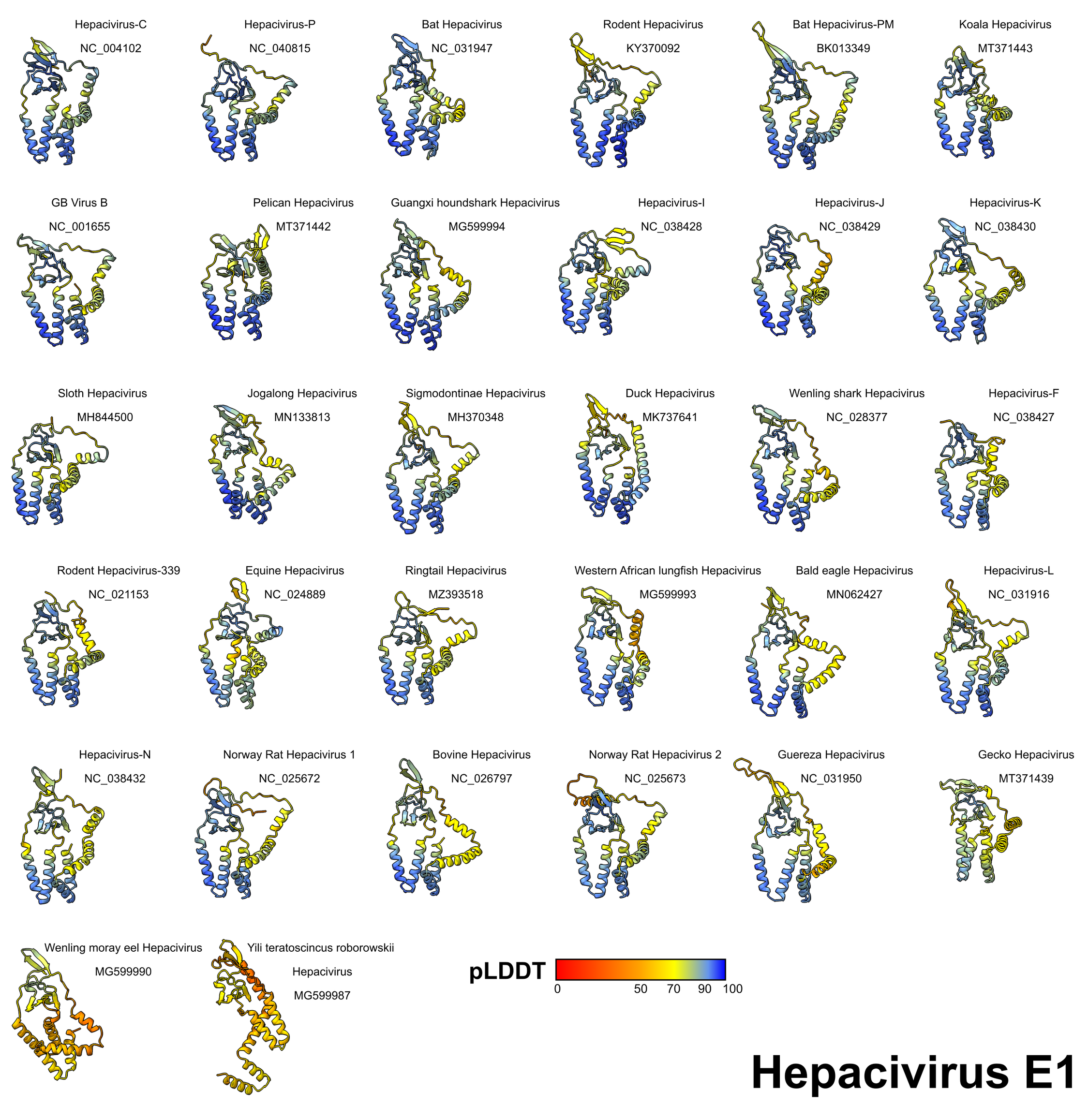

Supplement: S4 Fig — Residues are colour coded by pLDDT prediction confidence. Models are arranged in order of descending prediction confidence. Further description can be found in S1 Text. (TIF) [file pbio.3002174.s007.tif]

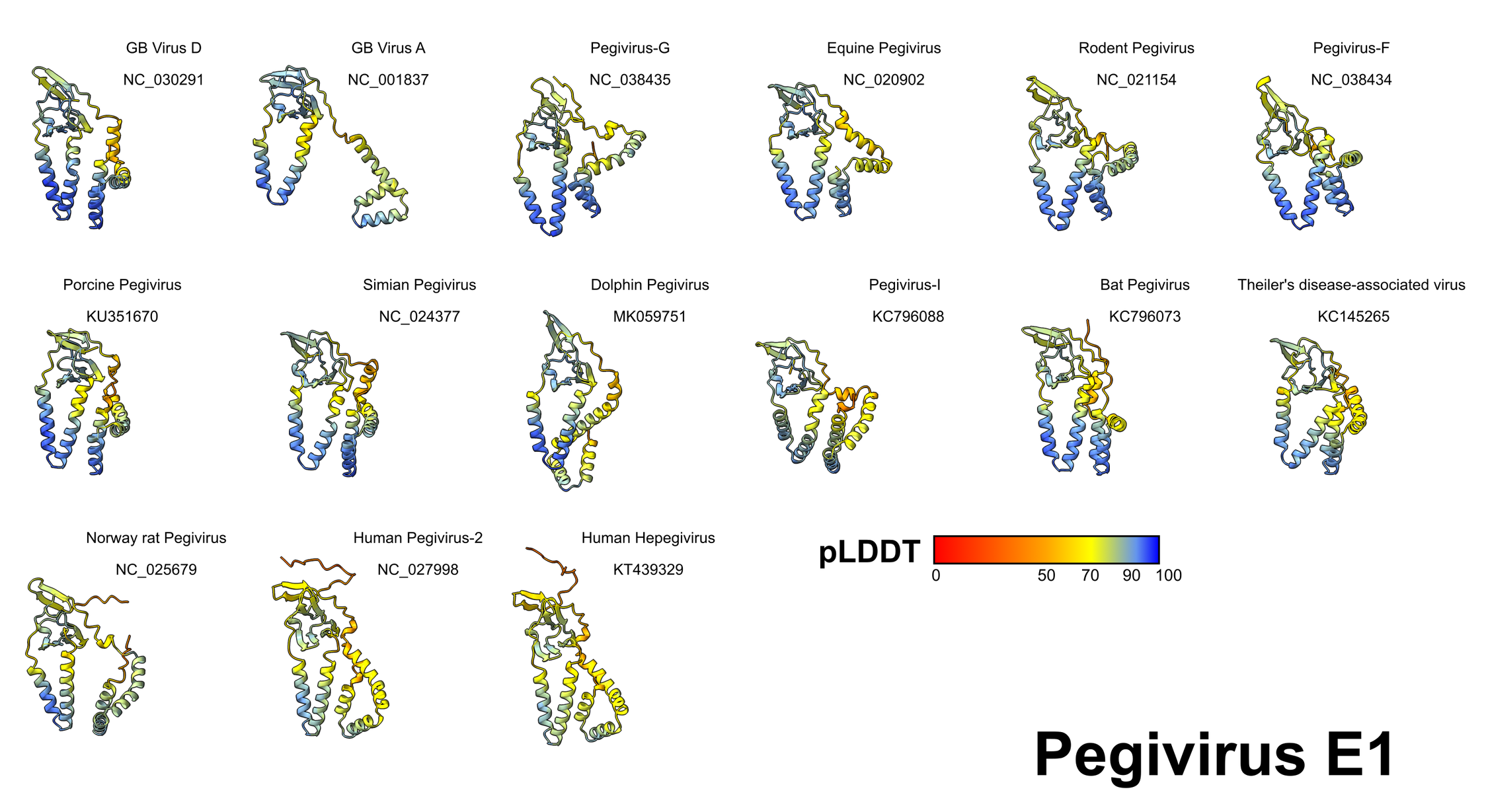

Supplement: S5 Fig — Residues are colour coded by pLDDT prediction confidence. Models are arranged in order of descending prediction confidence. Further description can be found in S1 Text. (TIF) [file pbio.3002174.s008.tif]

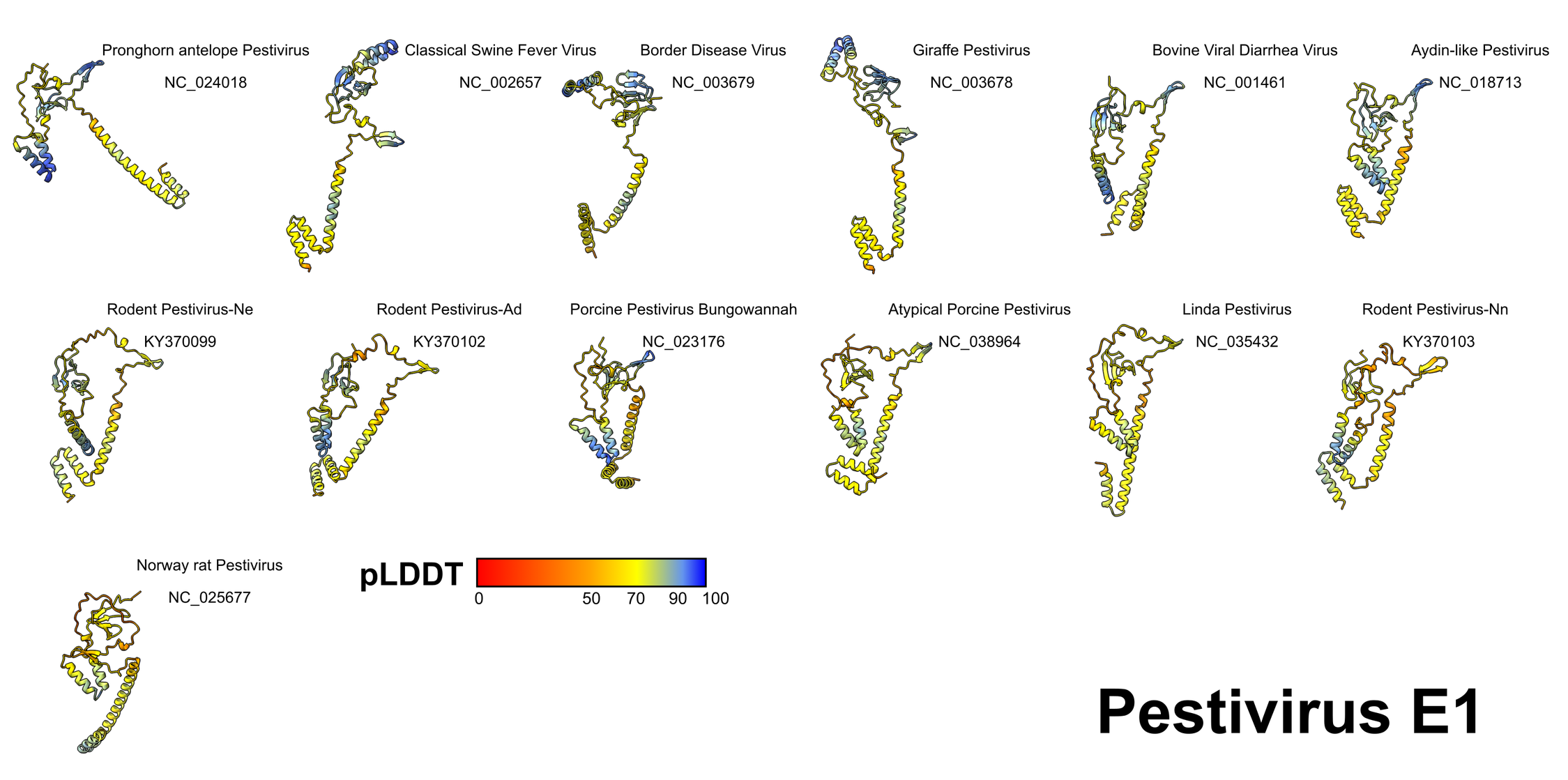

Supplement: S6 Fig — Residues are colour coded by pLDDT prediction confidence. Models are arranged in order of descending prediction confidence. Further description can be found in S1 Text. (TIF) [file pbio.3002174.s009.tif]

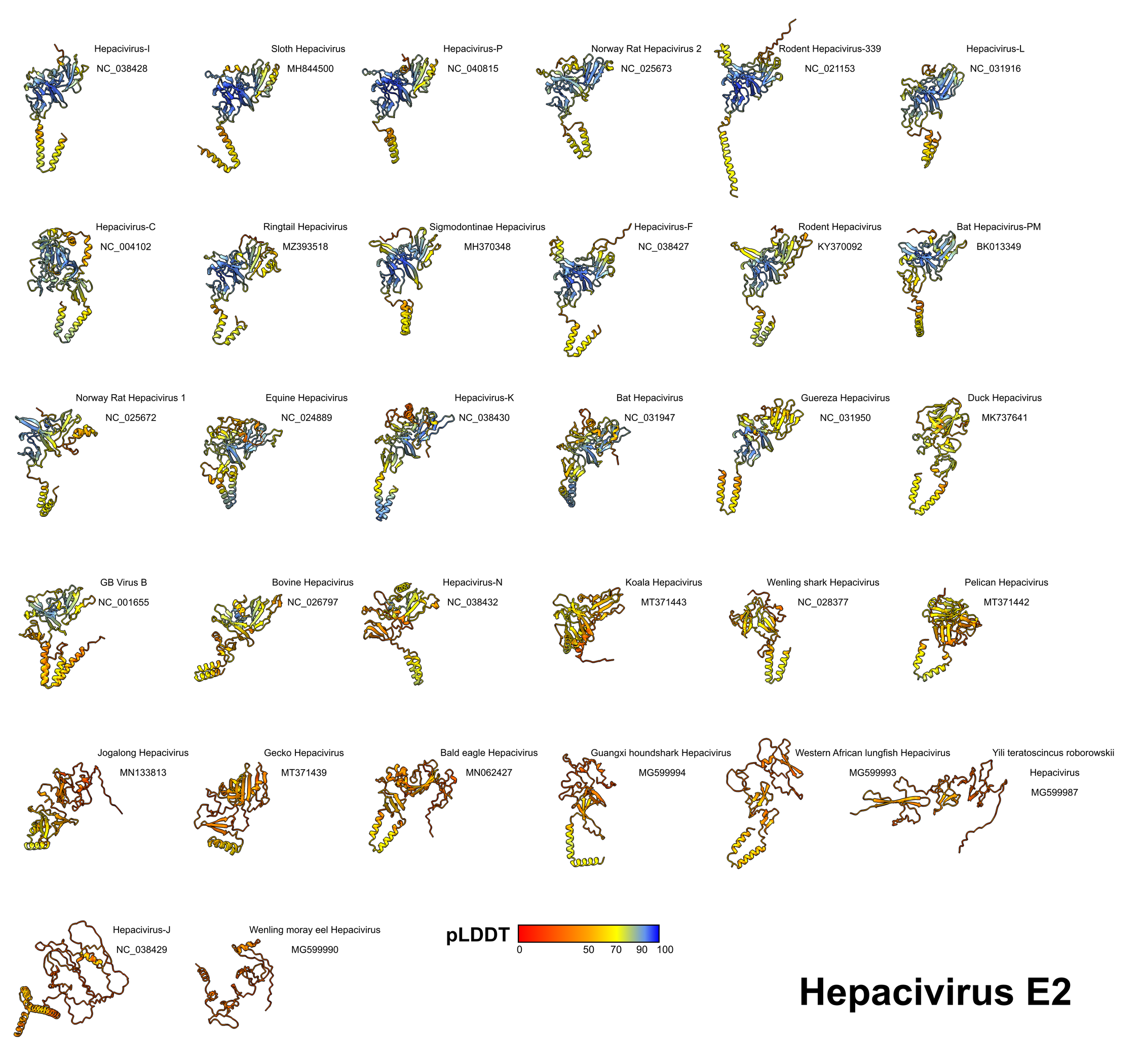

Supplement: S7 Fig — Residues are colour coded by pLDDT prediction confidence. Models are arranged in order of descending prediction confidence. Further description can be found in S1 Text. (TIF) [file pbio.3002174.s010.tif]

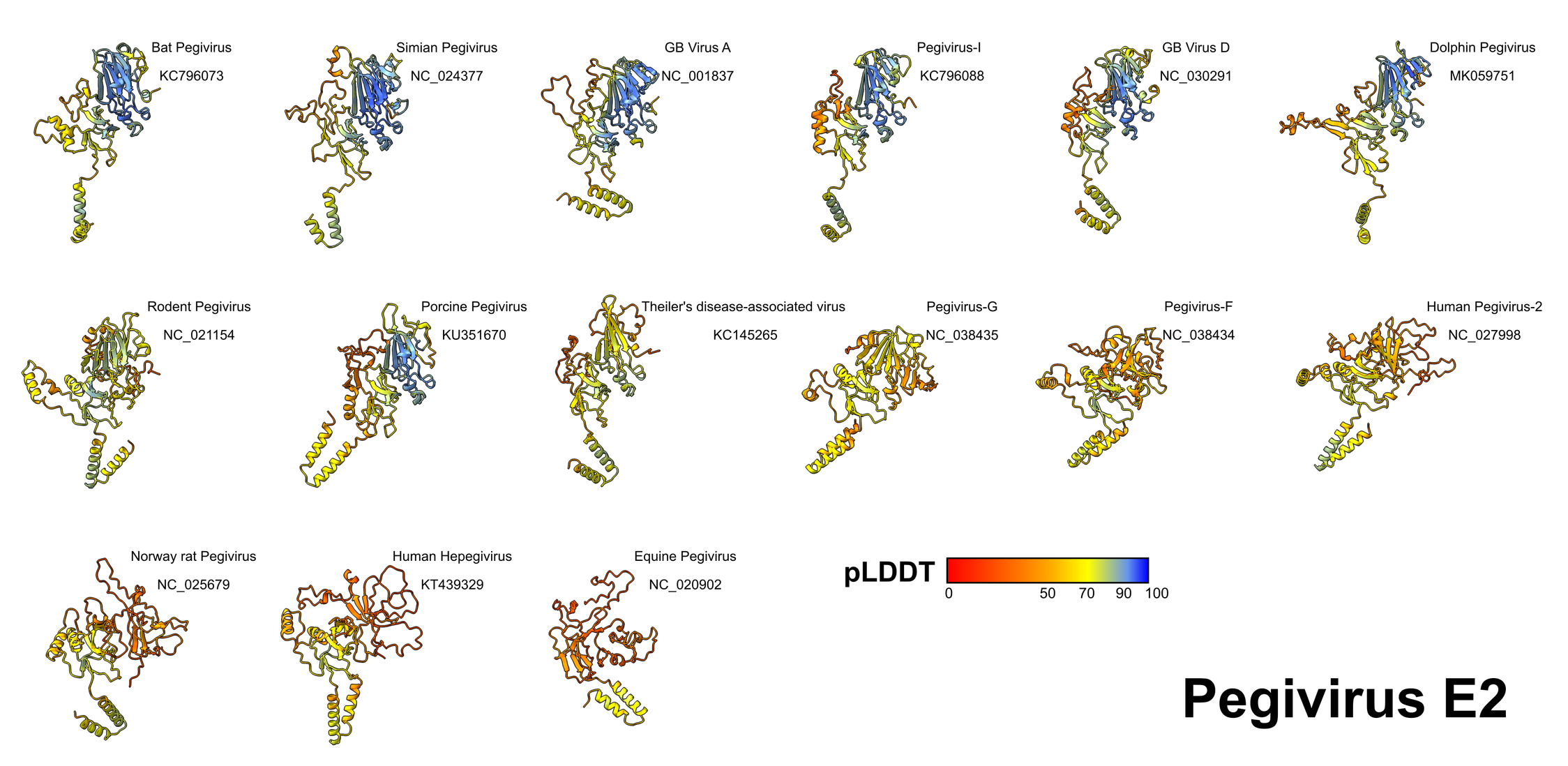

Supplement: S8 Fig — Residues are colour coded by pLDDT prediction confidence. Models are arranged in order of descending prediction confidence. Further description can be found in S1 Text. (TIF) [file pbio.3002174.s011.tif]

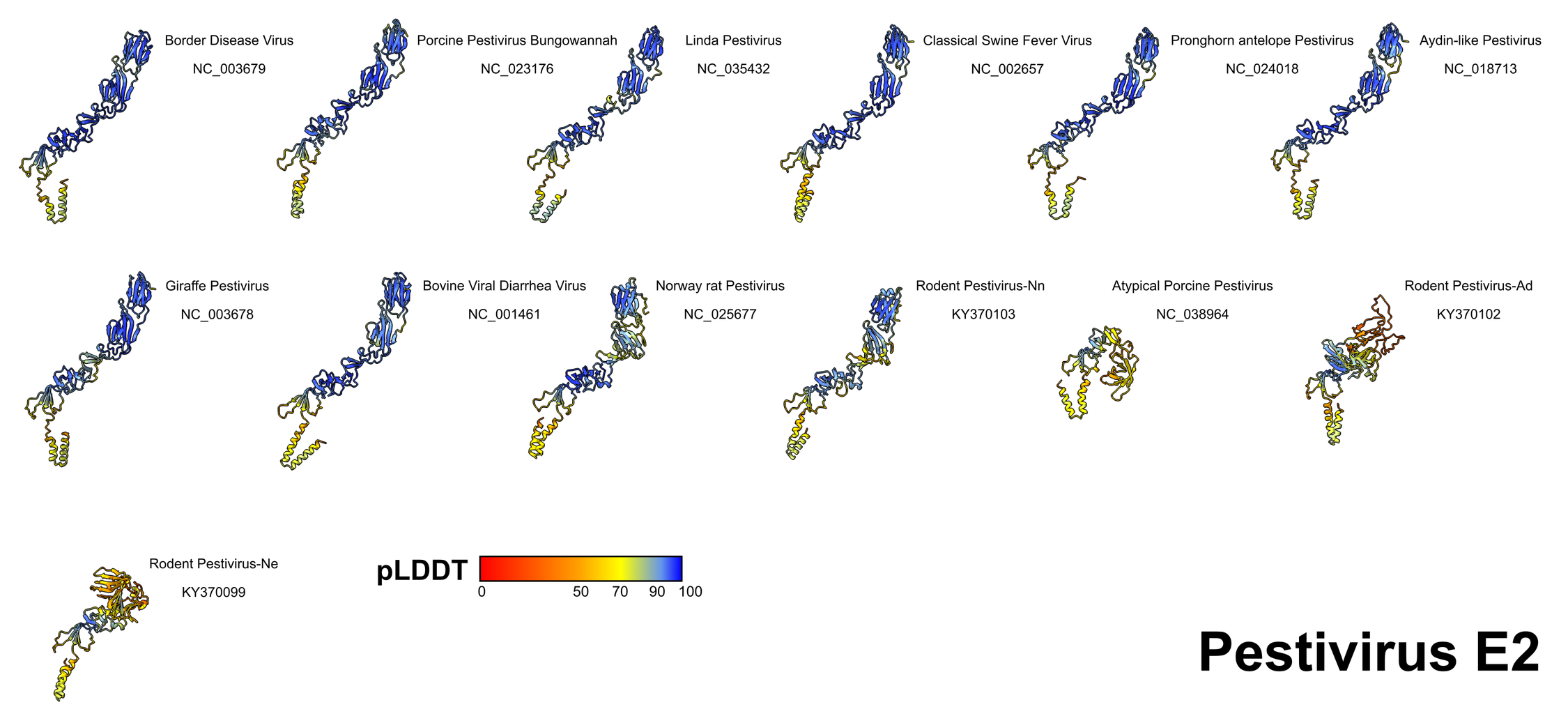

Supplement: S9 Fig — Residues are colour coded by pLDDT prediction confidence. Models are arranged in order of descending prediction confidence. Further description can be found in S1 Text. (TIF) [file pbio.3002174.s012.tif]

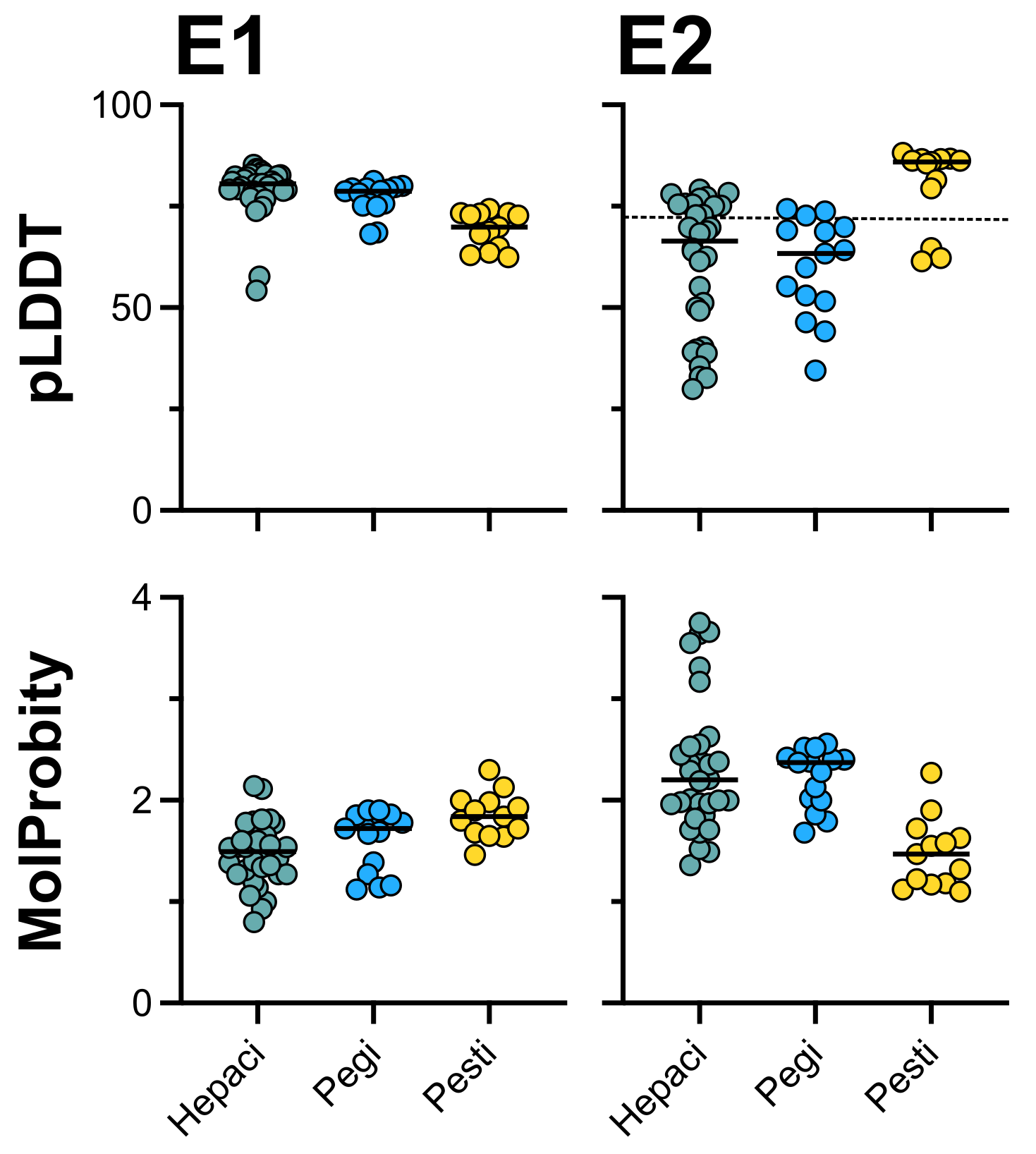

Supplement: S10 Fig — pLDDT prediction confidence (upper plots) and MolProbity scores (lower plots) for E1 and E2 models from diverse Hepaci-, Pegi-, and Pestiviruses. Each data point represents an individual viral species, n = 32, 15, and 13, respectively. Dashed line on upper right plot indicates pLDDT = 70 cutoff that was used to select viruses for modelling of E1E2 complexes. Underlying numerical data are available in S1 File. Further description can be found in S1 Text. (TIF) [file pbio.3002174.s013.tif]

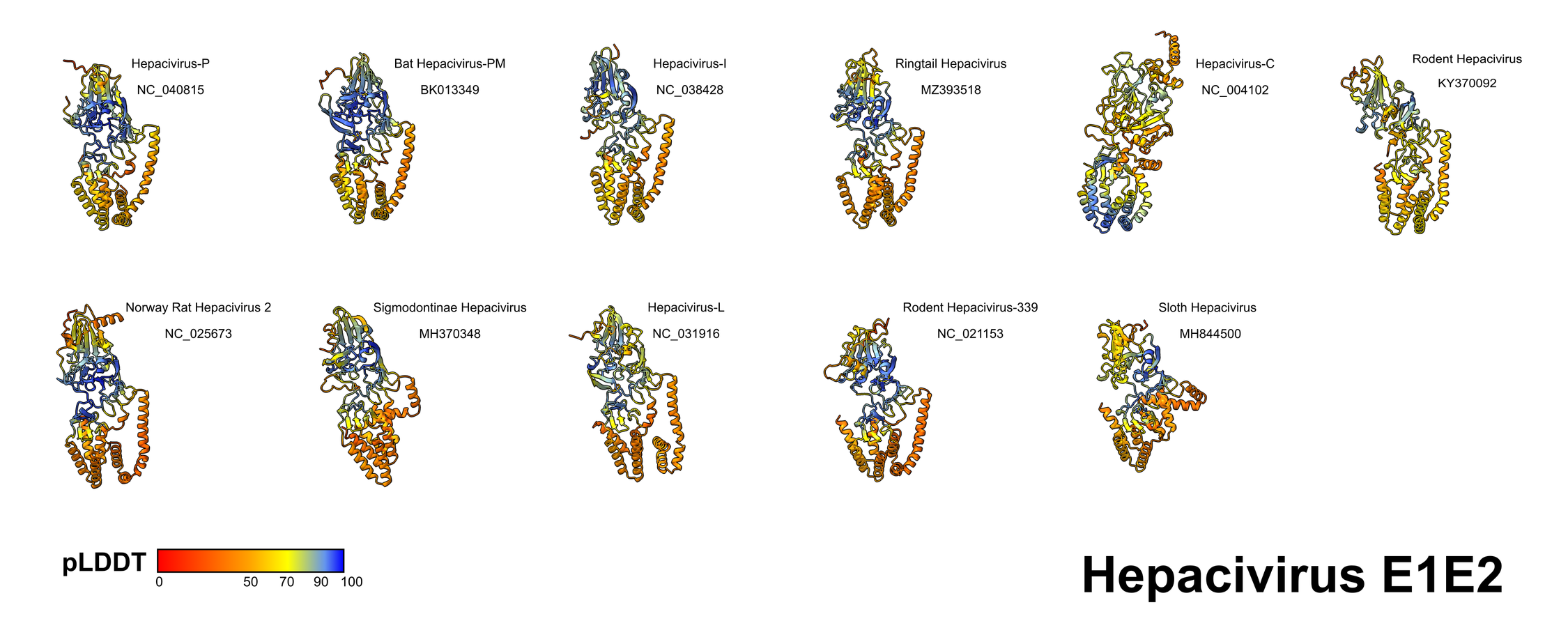

Supplement: S11 Fig — Residues are colour coded by pLDDT prediction confidence. Models are arranged in order of descending prediction confidence. Further description can be found in S1 Text. (TIF) [file pbio.3002174.s014.tif]

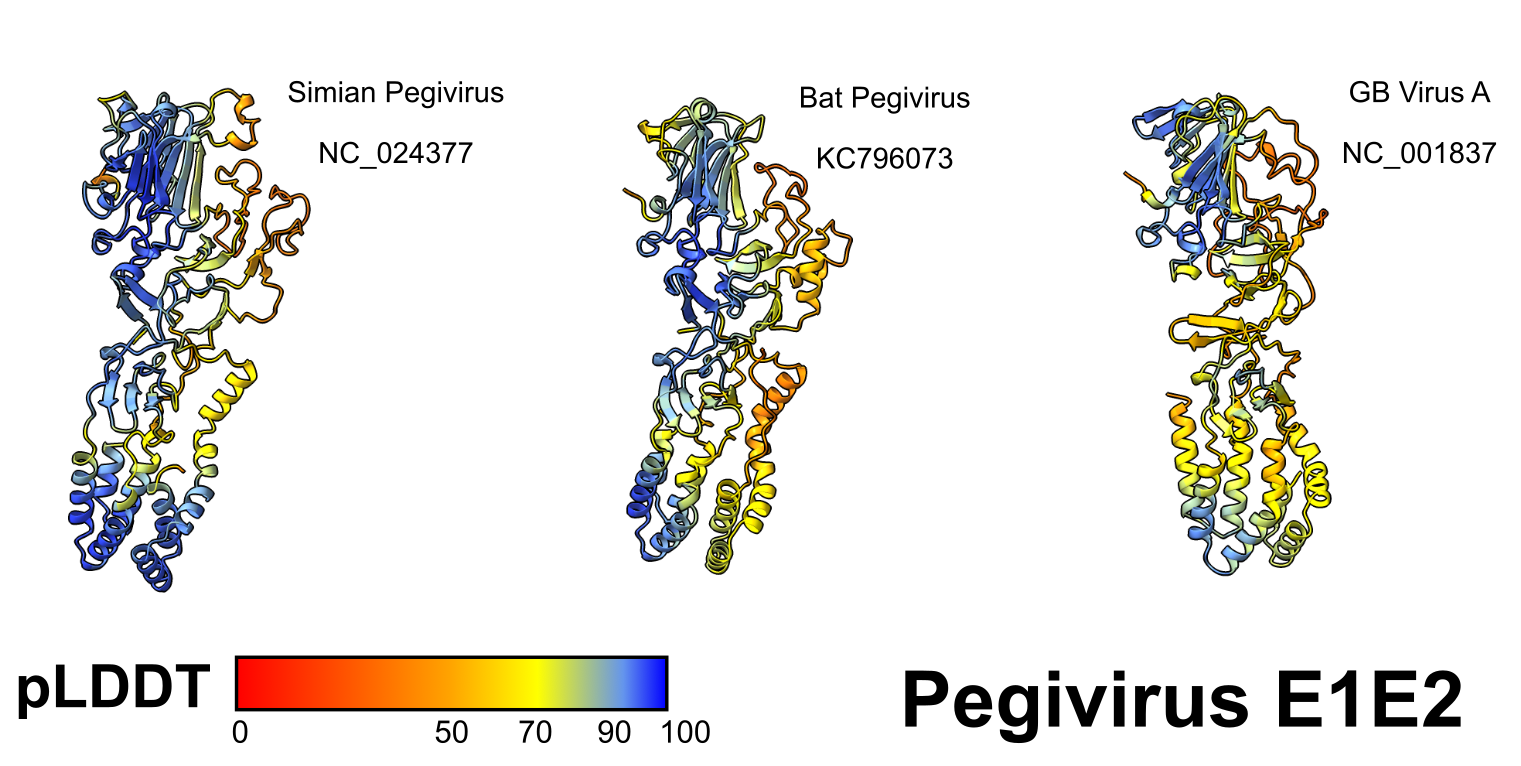

Supplement: S12 Fig — Residues are colour coded by pLDDT prediction confidence. Models are arranged in order of descending prediction confidence. Further description can be found in S1 Text. (TIF) [file pbio.3002174.s015.tif]

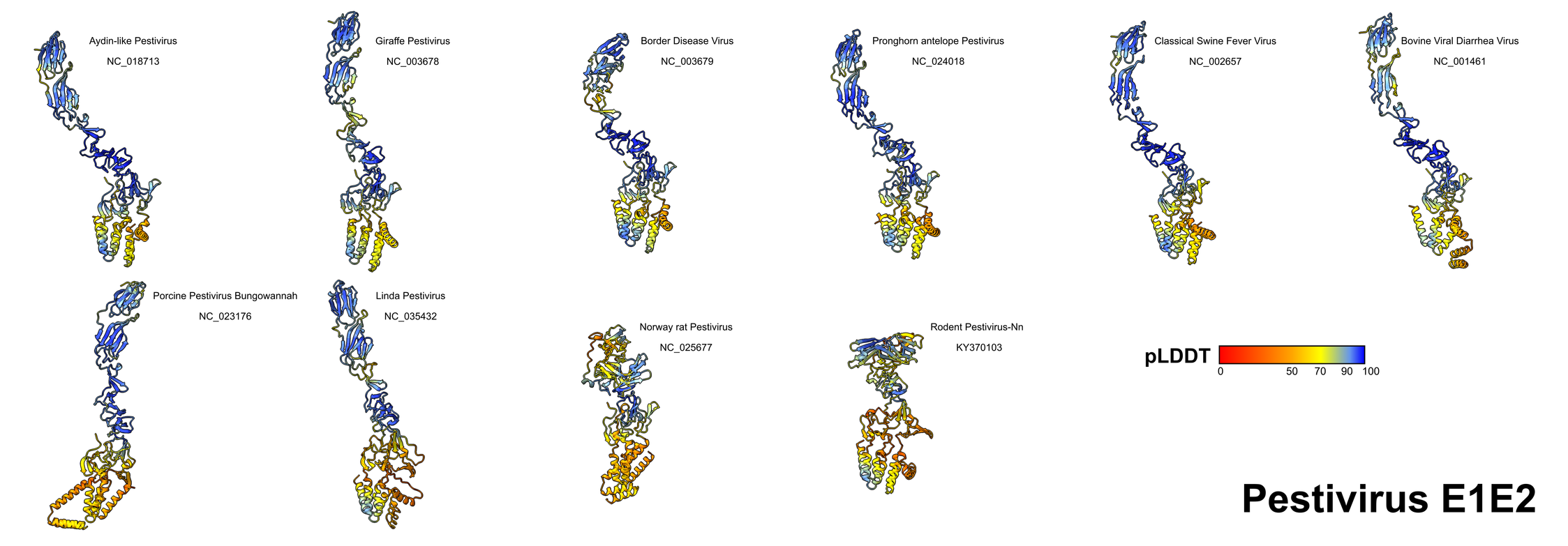

Supplement: S13 Fig — Residues are colour coded by pLDDT prediction confidence. Models are arranged in order of descending prediction confidence. Further description can be found in S1 Text. (TIF) [file pbio.3002174.s016.tif]

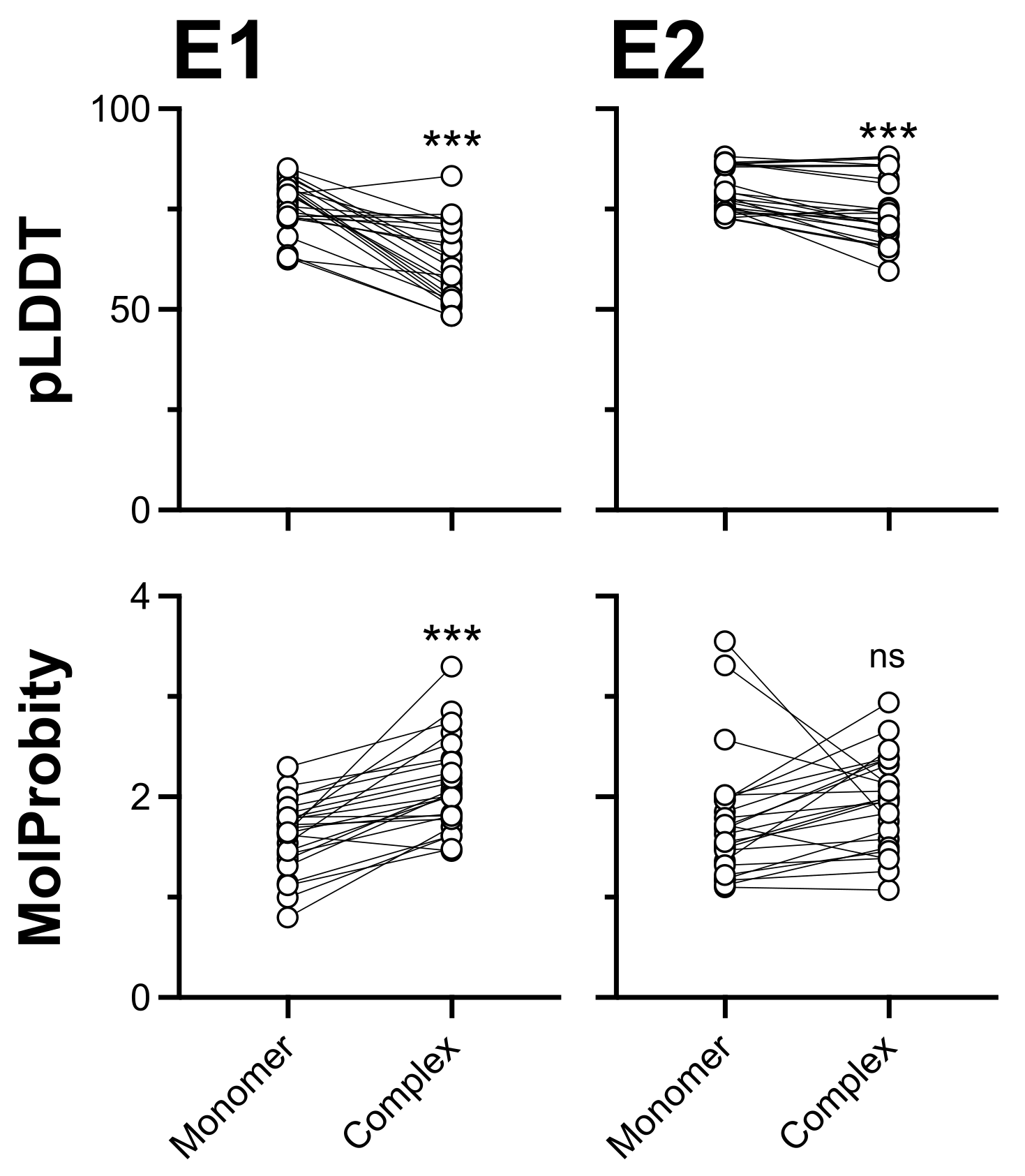

Supplement: S14 Fig — pLDDT prediction confidence (upper plots) and MolProbity scores (lower plots) for E1 and E2 modelled as a monomer or in a complex. Each data point represents an individual viral species, n = 24. Asterisks indicate degree of statistical significance (t test). Underlying numerical data are available in S1 File. Further description can be found in S1 Text. (TIF) [file pbio.3002174.s017.tif]

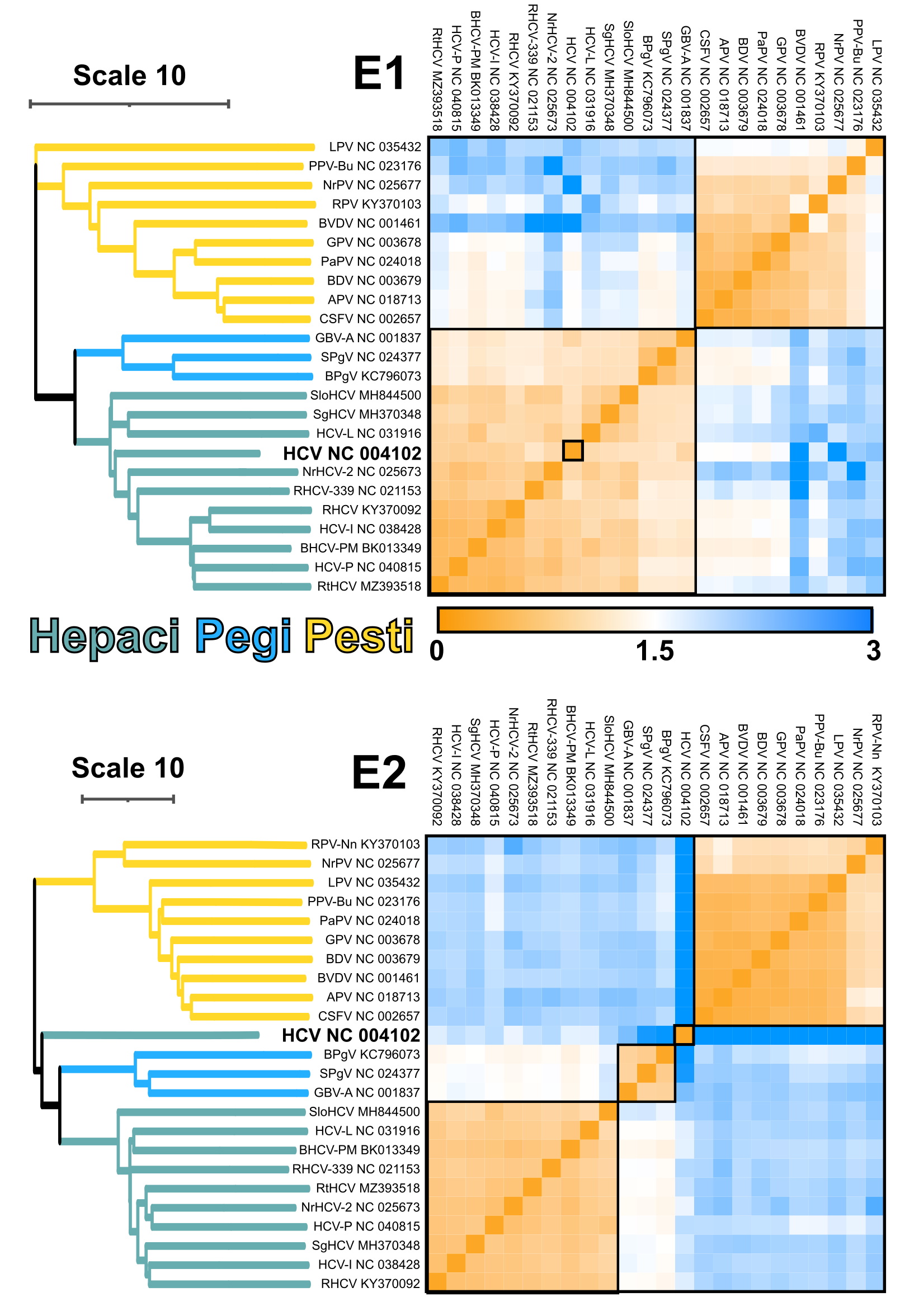

Supplement: S15 Fig — All against all comparison was performed on either E1 or E2 (derived from E1E2 complex models) using the DALI server. The heat map (right) provides pairwise distances indicating structural similarity (orange denoting low distances and, therefore, high similarity). Structures are also clustered by similarity, as denoted by the dendogram (left). Boxes are drawn to group structures with high similarity. The position of HCV is additionally highlighted. Underlying numerical data are available in S1 File. Further description can be found in S1 Text. (TIF) [file pbio.3002174.s018.tif]

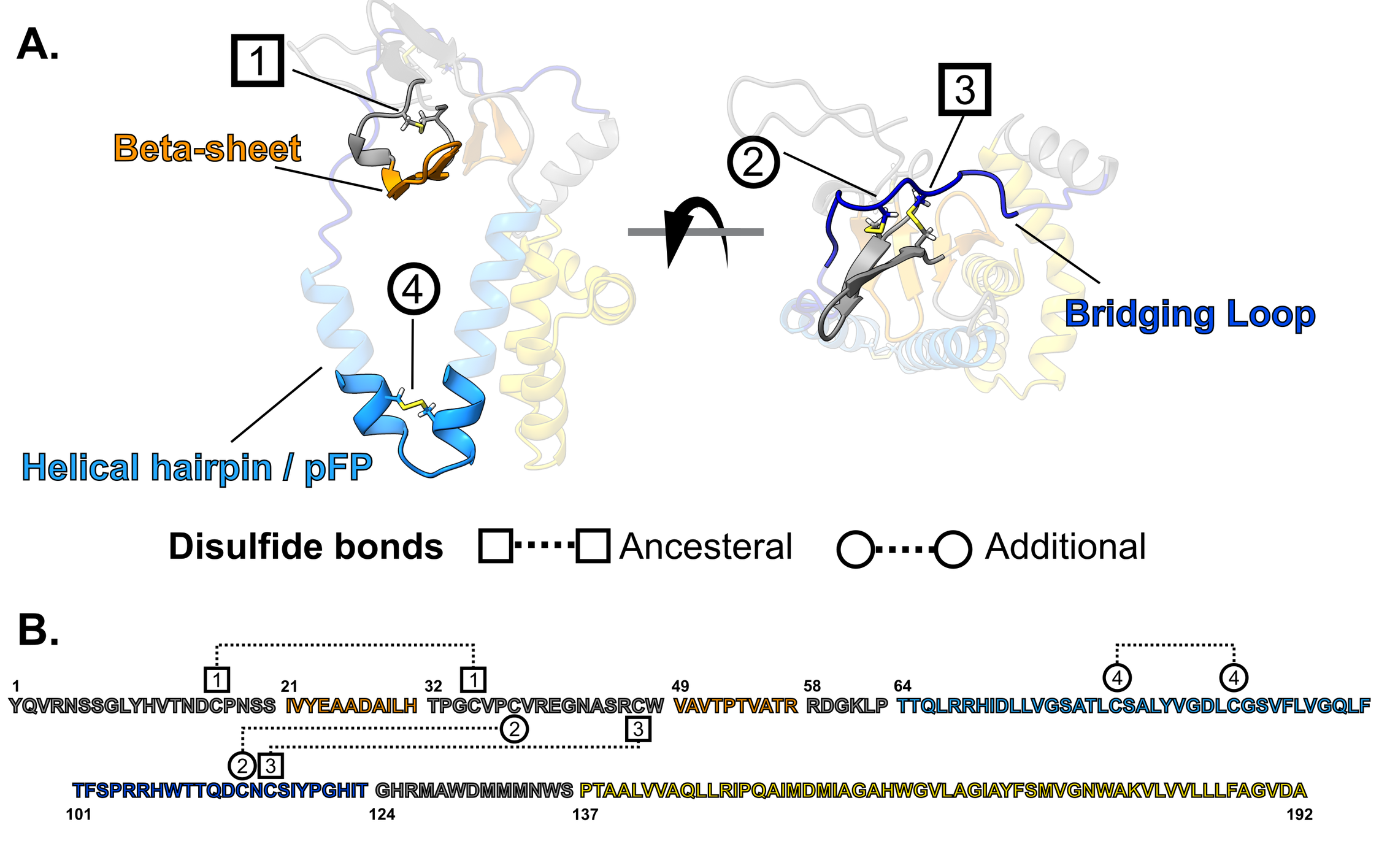

Supplement: S16 Fig — (A) Ribbon diagrams illustrating the location of the ancestral and additional disulfide bonds described in Fig 2. (B) Linear representation of HCV E1 sequence (H77 isolate), annotated with the position of each disulfide bond. (TIF) [file pbio.3002174.s019.tif]

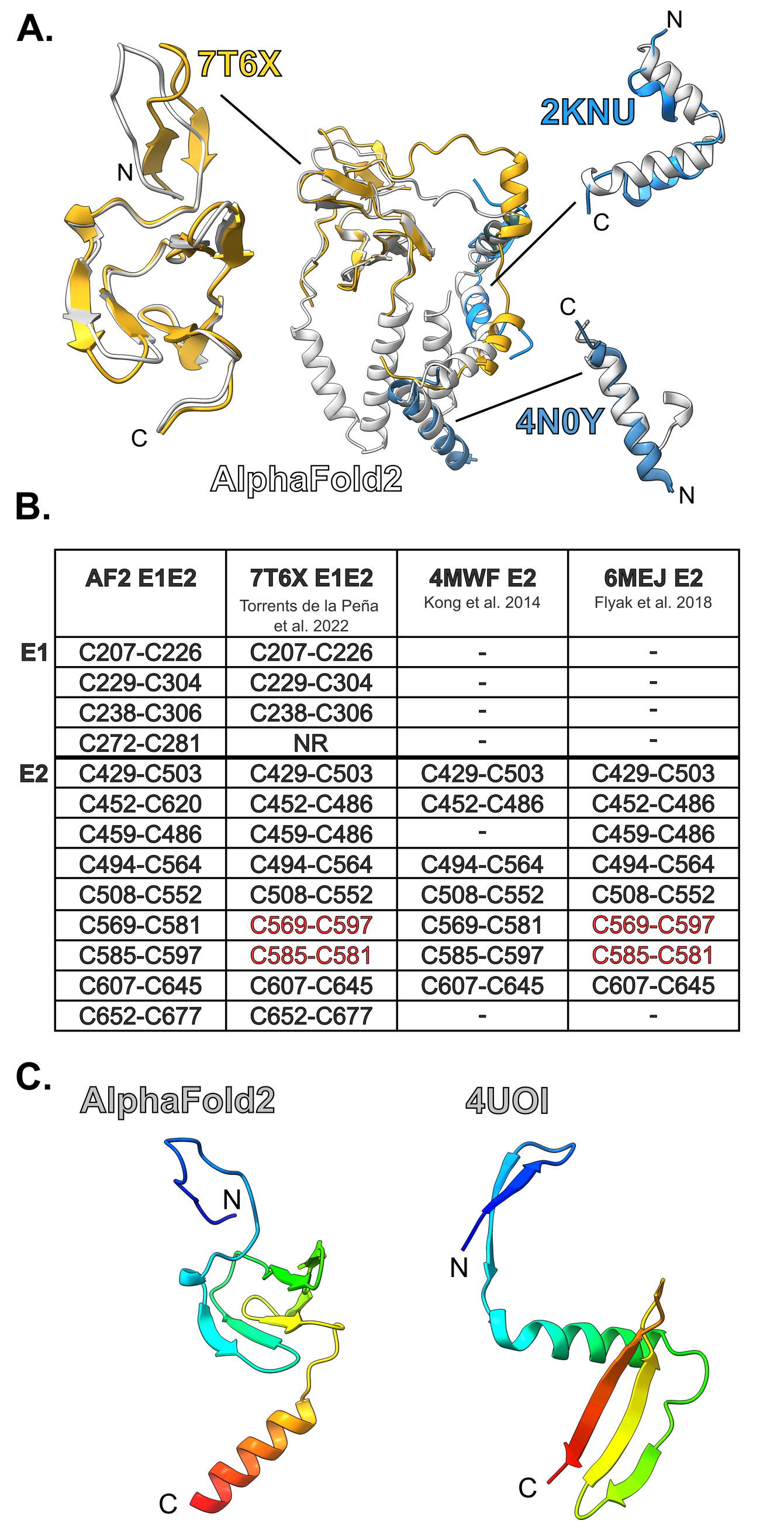

Supplement: S17 Fig — (A) E1 model compared to cognate cryoEM (PDB 7T6X) and peptide structures (2KNU and 4N0Y); superposition was achieved by alignment of structure and sequence. (B) Comparison of predicted E1E2 disulfide bonds with 3 experimentally determined structures. Bonds in black text are in agreement; red text indicates disagreement. Dashes indicate disulfides that were absent from the protein construct; NR, not resolved. (C) The N-terminal portion of AlphaFold HCV E1 model and the cognate crystal structure (PDB:4UOI). Further description can be found in S1 Text. (TIF) [file pbio.3002174.s020.tif]

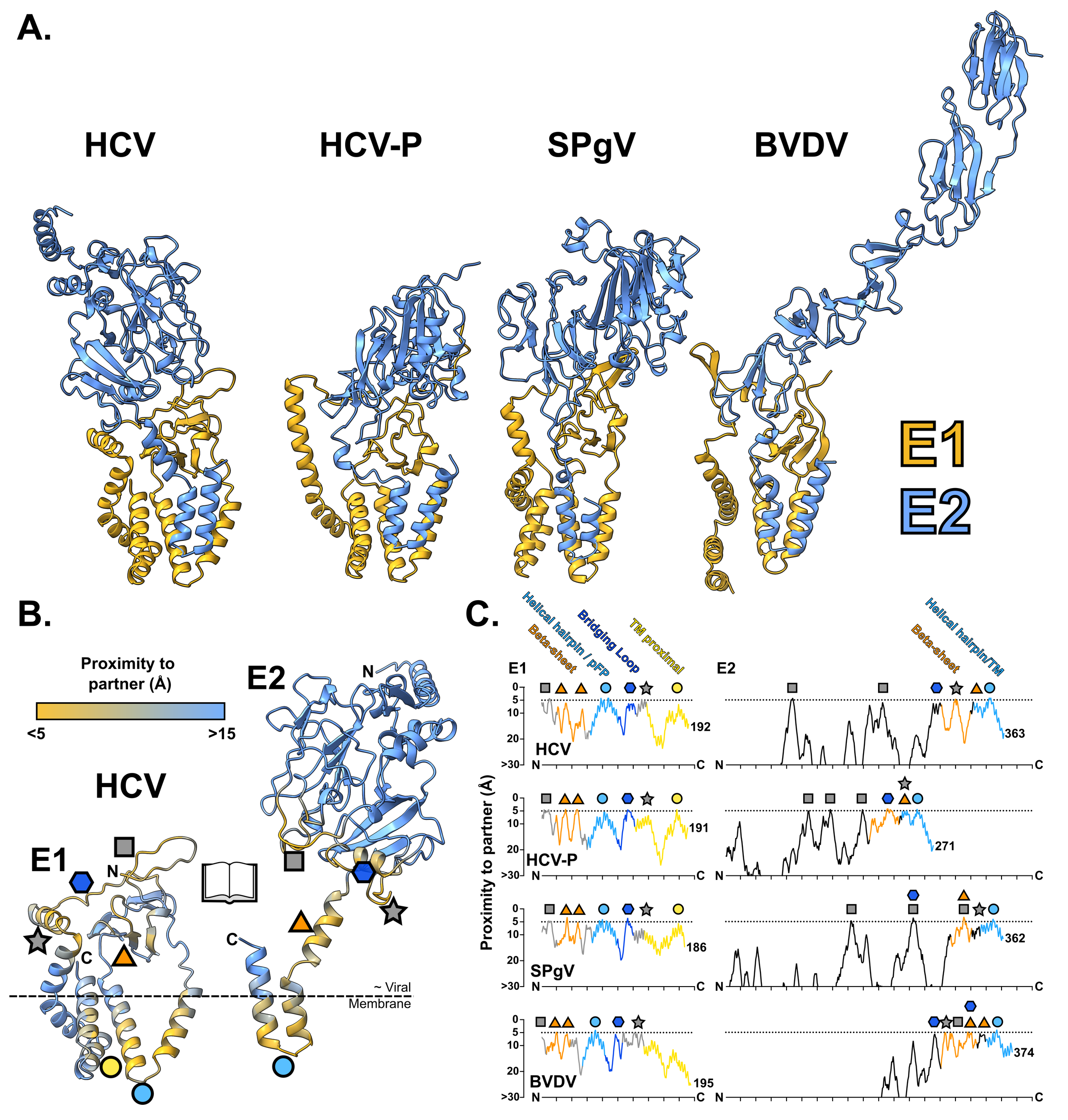

Supplement: S18 Fig — (A) Ribbon diagrams of the E1E2 complex from HCV, HCV-P, SPgV, and BVDV. (B) HCV E1E2 complex interaction interface in an “open book” representation. Residues are colour coded by their shortest distance to the partner protein (C⍺ to C⍺) as indicated by the colour key. Symbols annotate contact sites between E1 and E2. The approximate location of the outer leaflet of the viral membrane is inferred by the positions of the E1 and E2 transmembrane domains. (C) Plots provide E1 and E2 per residue shortest distance to partner protein (i.e., proximity of any given E1 residue to E2, and vice versa) for HCV, HCV-P, SPgV, and BVDV. Symbols relate to sites of contact, as annotated in (B). Lines are colour coded by structural features as in main text 2A (E1) and 3C (E2). Underlying numerical data are available in S1 File. Further description can be found in S1 Text. (TIF) [file pbio.3002174.s021.tif]
