# Supplementary material for: Structures of the Hepaci-, Pegi-, and Pestiviruses envelope proteins suggest a novel membrane fusion mechanism
Source: PLoS Biol. 2023 Jul 11;21(7):e3002174. doi: 10.1371/journal.pbio.3002174 (PMC10335668; doi:10.1371/journal.pbio.3002174)
Supplement: Attachment — Submitted filename: Responses.pdf [file pbio.3002174.s022.pdf]

Comments are provided, verbatim, in **black** text.
Responses are in **blue** text.

**Editorial Comments**

In particular, it is important that you tone down any claims of fusion mechanism, the manuscript would need to add more discussion along potential fusion models and it would need to be refocused to discuss the issues raised by reviewer #2.

We have made edits throughout the manuscript and added significant discussion around potential mechanistic insights in line with the suggestions of both reviewers 1 and 2.

Regardless of the method selected, please ensure that you provide the individual numerical values that underlie the summary data displayed in the following figure panels as they are essential for readers to assess your analysis and to reproduce it: Figure 1BDE, 2D, 3E, and Supplementary Figure S1BCD, S3, S10, S14, S15, S17C. NOTE: the numerical data provided should include all replicates AND the way in which the plotted mean and errors were derived (it should not present only the mean/average values). Please also ensure that figure legends in your manuscript include information on where the underlying data can be found, and ensure your supplemental data file/s has a legend. Please ensure that your Data Statement in the submission system accurately describes where your data can be found.

We have provided all underlying numerical data in Supplementary File 1 and have altered the manuscript to reflect this.

We suggest a change in the title to tone down the claims reagrding the mechanism. We suggest: "Common origin and structural features of the envelope proteins of Hepaci, Pegi and Pestiviruses suggests a new membrane fusion mechanism" or "Common origin and structural features of the envelope proteins of Hepaci, Pegi and Pestiviruses suggest a potentially new membrane fusion mechanism".

We gave this some careful consideration and would like to propose the title: “Structures of the Hepaci-, Pegi- and Pestiviruses envelope proteins suggest a novel membrane fusion mechanism.” We would prefer not to mention ‘origin’ in the title as I don’t believe our work provides a very clear picture of this.

**Reviewer one.**

1. My major concern is that the authors state a number of times (even in the title) that this identifies a "novel membrane fusion mechanism". While this may turn out to be true, this study does not show anything regarding fusion mechanisms. I would say this study identifies the "potential" for a novel membrane fusion mechanism and even new class of fusion glycoprotein. Or maybe "evidence of a unique viral membrane fusion mechanism" . It is clear from your modeling these don't look like the other fusion proteins. However, like the authors said there are many caveats here (pre- vs. post-fusion structures, limited experiments). Therefore, I suggest the authors reword these phrases to reflect their conclusions. Regardless, highlighting that these may be a new class of fusion protein is really cool! If the authors want to go down the road of fusion, can the authors speculate on a model of how these proteins would initiate membrane fusion? There is significant work done on HCV pH-dependent entry. Can you use your modeling to fit into these studies? In addition, there are proposed pestivirus models of fusion (PMID: 23569276). How do your models fit in there? Are there flexible domains that could move to drive fusion? I think speaking more about fusion mechanisms will be required to keep the "novel membrane fusion mechanism" angle.

Thanks to the reviewer for their valuable comments. We agree that without further mechanistic studies it is not possible to definitively conclude that we have identified a novel fusion mechanism. Moreover, we acknowledge that we had not done enough to place our work in the context of previous functional studies of HCV and BVDV entry.

To address this we have made edits throughout the manuscript (including the title) to weaken our statements around identification of a novel fusion mechanism. We have also made extensive additions to the discussion to consider our work in the context of what is already known of HCV/BVDV entry. In

particular: reconciling our work with prevoiusly proposed models of HCV/BVDV fusion; consideration of which protein, E1 or E2, is likely to be the primary fusogen; and how our work relates to current understanding of the molecular cues that regulate HCV/BVDV entry. This can be found from line 225 to 270. We also consider how our E1E2 predicted structures relate to the reported E2-E2 dimers in BVDV (Lines 187-190). The edits have given our work considerably more depth and we thank the reviewer for these suggestions.

2. A more clarity comment and I'm sorry if I missed it. Can you provide on Figure 1 and 2 where the fusion peptide is located? Also, in figure 2A. How many transmembrane domains are present? By this schematic, it looks like the blue helices are TMD too. Is that true? Can you modify to clear that up?

We agree that this simply wasn't clear enough and we have made edits throughout the text and figures to reiterate which region corresponds to the putative fusion peptide; this also clarifies the confusion around Figure 2A. Edits can be found to Figure 1,2,3 and S18.

**Reviewer two.**

**Major**

1. This last point concerns the trans-membrane (TM) regions. Because most of the viral fusion proteins are type 1 transmembrane proteins anchored at the viral surface by a single TM segment at the C-terminal end, it had been expected that E1 and E2 would be the same. The Flaviviruses are actually an exception, because the envelope proteins in this genus have an alpha-helical segment instead of a single TM segment at the C-terminal end, unlike their counterpart in alphaviruses and bunyaviruses. The AlphaFold predictions indicate instead that E1 and E2 span the membrane multiple times. This is very clear in Figure 4A, showing the TM helical bundle anchored in the membrane, and the beta-sheet and additional extensions projecting out of the bundle to the extra-viral space. The authors, however, appear to overlook this important result. Furthermore, as they show in Figure 4D, the ancestor of these proteins appears to have been a simple protein with a small beta sheet followed by a hydrophobic alpha-helical hairpin, which can be safely interpreted as a TM anchor. E1 and E2 thus appear to have undergone different paths during their subsequent evolution, from a potential homodimer to then a heterodimer (upon gene duplication). E1 acquired an additional alpha-helical TM hairpin and peri-membrane-region at its C-terminal half, and E2 evolved host-specific extensions and additions to the initial beta-sheet, to the point that the beta sheet element is altogether absent in the pestiviruses.

We are grateful to Reviewer 2 for their insights and suggestions on our work. Comments numbered 1, 3 and 4, here, all pertain to the unusual features of E1E2 and how they can, and cannot, be understood in the context of other viral fusion proteins. Our revised discussion (Lines 225-270) tackles all of the major points raised in comments 1,3 and 4 – in particular: whether E1E2 fits our current understanding of viral fusogens; the apparent presence of extensive transmembrane/membrane-associated regions, as seen in other viruses such as HBV; and whether we need to consider radically different mechanisms of fusion. Thanks to the reviewer; we believe this has strengthened our work significantly.

2. Furthermore, the distance in the membrane spanned by the TM segments is shorter than a normal lipid bilayer spanning segment, but this is also the case for the alpha-helical TM hairpin of their fusion protein. The reason is that the bilayer in the viral particle is highly constrained and very thin in the areas where the TM segments are inserted, as shown by the cryo-EM structures. For the hepatitis C virus, in contrast, it has been proposed that the glycoproteins are anchored in a lipid structure similar to that of lipid droplets, which display a glycerophospholipid monolayer surrounding t triglyceride-rich core. The dimension of the structure displayed in Figure 4D would be an excellent fit on the monolayer in this model

We agree that the length of the transmembrane domains is an interesting observation and may correlate with the architecture of HCV virions. However, E1E2 are able to function on pseudotype viruses (which have a conventional lipid bilayer) and there isn't evidence that Pegi- or Pestiviruses associate with lipd

droplets/lipoproteins. Consequently, in the absence of more information in this area, we have chosen not to speculate about this in the text.

3. (Related to 1.) The original postulate that the first hydrophobic segment of E1 could constitute a fusion loop was done at a time where no structural data were available, and when it was considered that hepaciviruses, pegiviruses and pestivirus would function similarly to the fusion proteins of viruses that had already been characterized, and which all feature a fusion loop (or fusion peptide) that inserts into the target membrane during a conformational change. But there are other viruses, such as the hepadnaviruses (HBV, for instance), the envelope protein of which has four TM segments and no predicted fusion loop or peptide. Or the poxviruses, which have a membrane fusion complex composed of at least 10 different proteins, all with transmembrane regions. It is evident that these viruses cannot induce membrane fusion using the mechanism of those for which the structural studies are available. This study therefore opens the door for beginning to understand the mechanism of fusion developedby them. It could be a concerted action between the fusion proteins, not necessarily the action of one of the two partners of the heterodimer, like in the class II fusion proteins.

See response to comment 1.

4. (Related to 1.) In my view, therefore, the authors are right to discuss that E1 and E2 must display a different mechanism to induce fusion than the flaviviruses, but they fail to see that they have provided crucial evidence to lump these three genera into a broader context of viruses having multiple TM segments and for which the mechanism of action is not understood. And here is where I see the true value of this paper. It is therefore important that the authors revisit the interpretation of their results, which are indeed major.

See response to comment 1.

**Minor**

1. Please provide more background concerning HCV-C, HCV-P, etc. The average reader of PLoS biology is most likely not a specialist in hepatitis C virus, and this terminology is certainly unfamiliar.

We have altered and simplified labels throughout the text and figures (e.g. refer to HCV instead of HCV-C) and added host species information to Figure 1 for further clarity. We also include Supplementary File 2 which outlines precisely which viruses have been used and provides the name abbreviations used in the text.

2. Maybe draw the disulfide bonds as green sticks in the cartoons of Fig. 2B

We have chosen not to do this due to considerations for color-blind individuals. We have, however, added an additional supplementary figure (S16) that further clarifies the location of the disulfide bonds on the 3D structure of HCV E1.

3. Line 30: please add bunyaviruses to the list of viruses with class II fusion proteins.

We have made this edit and added citations appropriately (Line 32).

4. Line 33: "eliminated any genetic homology". The term "homology" is incorrectly used here. What the authors mean is that the homology cannot be detected by sequence comparisons, as during divergent evolution the proteins have lost all sequence similarity. But their homology can still be detected by structural comparisons. So, what the authors mean is that evolutionary divergence has eliminated any sequence similarity that could allow to detect homology. This is also true for the supplementary text, where they say: "this revealed low homology across most of the genome in every genus". This sentence does not make sense, as obviously they are clear homologs (in line 73 of the supplementary material). The same is true for the sentence in line 144, where the authors mean "the high degree of structural similarity". Homology cannot have a high or low degree: it is binary. Two proteins are either homologous or they are not, what differs is the degree of diversity from their common ancestor.

We have made edits throughout the main and supplementary text to ensure that we are using ‘homology’ and ‘similarity’ correctly. Thank you for the advice on this.

5. Line 50: the minor glycoprotein E1. It is not a "minor" protein, as it is produced in stoichiometric amounts with E2.

We removed references to E1 as a ‘minor’ glycoprotein (Line 48-49)

6. Line 69: primary sequence similarity, not homology. Same for line 72: little/no sequence similarity, not homology.

See response to minor comment 4.

7. Line 186: should be "HCV-C E1E2"

Thank you, we have corrected this.

8. Legend to Fig. 3E: "Structures are shown in two opposite orientations". This is not clear: It seems to me that they are viewed rotated by 180 degrees about a vertical axis. It would be helpful to add a labelled rotation symbol in between the two views.

We have edited Figure 3 accordingly.

9. Figure 4B: Is this an "open book" representation of the complex shown in 4A? Please state so in the legend (maybe also provide an open book symbol, for clarity). Same for Fig. S17B.

We have edited Figure 4B and 17B and their respective legends.

10. Legend to Figure 4C: "Ribbon diagrams demonstrating the 'ancestral' E1E2 interface". I would say "illustrating" rather than "demonstrating the 'ancestral' E1E2 interface". Same for 4E.

Thank you, we have corrected this.

11. SM lines 114-115: "A simple visual examination of the Hepaci and Pegivirus E1 structures (Fig. S3 and 4)" Should be Fig. S4 and 5 instead?

Thank you, we have corrected this.

12. SM lines 130-131 "such that Pestivirus E1, form E1E2 complexes" there is a typo and an extra comma here, which makes the sentence difficult to read.

Thank you, we have corrected this.

13. SM line 214: typo in "Mutant E12 constructs"?

Thank you, we have corrected this.

14. It might make sense to try the HorA (http://prodata.swmed.edu/horaserver; doi: 10.1093/nar/gkp328) server to assess the homology of E1 and E2, and across the three genera being examined.

Thank you for this suggestion. We submitted our structures to the HorA server 4 weeks ago, however, the analyses are still running and it is not clear when they will complete. We will consider it for future investigations though.
